# The characteristics of circRNA as competing endogenous RNA in pathogenesis of acute myeloid leukemia

**DOI:** 10.1186/s12885-021-08029-7

**Published:** 2021-03-15

**Authors:** Siyuan Zhang

**Affiliations:** School of Medicine, Xi’an Jiaotong University, 76 Western Yanta Road, Xi’an, 710061 Shaanxi China

**Keywords:** Acute myeloid leukemia, CircRNA, Competing endogenous RNA

## Abstract

**Background:**

As one of the novel molecules, circRNA has been identified closely involved in the pathogenesis of many diseases. However, the function of circRNA in acute myeloid leukemia (AML) still remains unknown.

**Methods:**

In the current study, the RNA expression profiles were obtained from Gene Expression Omnibus (GEO) datasets. The differentially expressed RNAs were identified using R software and the competing endogenous RNA (ceRNA) network was constructed using Cytoscape. Functional and pathway enrichment analyses were performed to identify the candidate circRNA-mediated aberrant signaling pathways. The hub genes were identified by MCODE and CytoHubba plugins of Cytoscape, and then a subnetwork regulatory module was established.

**Results:**

A total of 27 circRNA-miRNA pairs and 208 miRNA-mRNA pairs, including 12 circRNAs, 24 miRNAs and 112 mRNAs were included in the ceRNA network. Subsequently, a subnetwork, including 4 circRNAs, 5 miRNAs and 6 mRNAs, was established based on related circRNA-miRNA-mRNA regulatory modules.

**Conclusions:**

In summary, this work analyzes the characteristics of circRNA as competing endogenous RNA in AML pathogenesis, which would provide hints for developing novel prognostic, diagnostic and therapeutic strategy for AML.

## Background

Acute myeloid leukemia (AML) is a type of malignant neoplasm characterized by clonal proliferation, abnormal growth and impaired differentiation of hematopoietic stem cell (HSC) in the hematopoietic system, in which immature myeloid cells infiltrate in the bone marrow, peripheral blood or other extramedullary tissue (such as the lymph nodes, spleen and central nervous system) [1–3]. With advance of molecular biology, the awareness and knowledge of tumorigenesis and development of AML is gradually growing. However, the outcome of AML patients remains dismal, with the 5-year overall survival (OS) of 40% for patients younger than 60 years and 10 ~ 20% for patients older than 60 years [4]. Hence, illustration of the molecular mechanism of pathogenesis underlying AML is crucial for seeking effective novel diagnostic and therapeutic targets.

Noncoding RNA (ncRNA), which is characterized by non-protein coding functional RNA, plays a crucial role in AML diagnosis, prognosis and treatment [5]. CircRNA, a large class of endogenous ncRNA, has a covalent closed loop building through a phosphodiester bond between their 3′ and 5′ ends, which is produced by an atypical splicing called back-splicing [6, 7]. Due to the circular structure of the circRNA, circRNA is speculated to be more stable than linear transcripts, showing higher median half-life and higher expression of some specific circRNAs than their linear transcripts [8]. Though ncRNA has been known for decades, the discovery of circRNA, which recognized recently, opens up new cognition of complex RNA-modulated gene expression [7]. The regulated function of circRNA is mainly based on the competing endogenous RNA (ceRNA) hypothesis which was proposed in 2011 [9]. The ceRNA hypothesis presents an idea that RNA transcripts communicate and regulate their respective expression levels through competing for binding microRNA response elements (MREs). CircRNA could function as miRNA sponges by binding to miRNA with MREs, which will prohibit mRNA degradation through miRNA-mediated pathways [6]. CircRNA could negatively regulates mRNA expression level as miRNA sponge by ceRNA network, and a growing number of evidences also have demonstrated this hypothesis [10]. For instance, one most well characterized circRNA is circRNA Cdr1as, which has 73 partially complementary binding sites for miR-7. Knocking down of Cdr1as from the mouse genome will downregulate the expression of miR-7 in the mouse brain, and thus, the mir-7 target genes (such as Fos) were upregulated in Cdr1as knockout mice [11]. CircPAN3 (hsa_circ_0100181) could mediate the chemoresistance of AML cells through circPAN3 − miR-153-5p/miR-183-5p − XIAP axis [12], and circDLEU2 (hsa_circ_0000488) could promote AML cell proliferation and inhibited cell apoptosis through circDLEU2 − miR-496 − PRKACB axis [13]. Nonetheless, more researches are still needed to explore the comprehensive role of circRNA as ceRNA in the pathogenesis of AML. In addition, circRNA could also be a potential biomarker for diagnosis and prognosis of AML [14].

In the current study, the expression profiles of circRNAs, miRNAs, and mRNAs in bone marrow of AML patients and healthy controls were collected from Gene Expression Omnibus (GEO) datasets. The differentially expressed mRNAs (DEmRNAs), differentially expressed miRNAs (DEmiRNAs), and differentially expressed circRNAs (DEcircRNAs) were identified using R software, and the interactions of circRNAs and miRNAs, and miRNAs and mRNAs were predicted using accepted algorithm. After that, the circRNA-miRNA-mRNA (ceRNA) network was established. To evaluate the main function that circRNA involved in the tumorigenesis of AML, gene ontology (GO) annotation and Kyoto Encyclopedia of Genes and Genomes (KEGG) pathway analyses were performed. Finally, The Cancer Genome Atlas (TCGA) database and Genotype-Tissue Expression (GTEx) database were used for validation and survival analysis of the hub genes. This study will take an attempt to provide a compressive insight for biomarker discovery, the pathogenesis of AML, and the development of the novel treatment strategy for AML.

## Methods

### Dataset collection

The circRNA, miRNA and mRNA expression profiles were downloaded from GEO (https://www.ncbi.nlm.nih.gov/geo/). The microarray data of circRNA were obtained from the GSE116617 [15] (including 8 patients and 4 healthy controls) and GSE94591 [16] (including 6 patients and 4 healthy controls). The miRNA expression data was obtained from the GSE142699 (including 24 patients and 24 healthy controls), and mRNA expression data was obtained from the GSE114868 [17] (including 194 patients and 20 healthy controls).

### Identification of differentially expressed circRNAs, miRNAs and mRNAs

The Bioconductor Limma [18] package (version 3.44.3) was used to identify DEcircRNAs, DEmiRNAs and DEmRNAs between AML patients and healthy controls. The significant DEcircRNAs (*P* < 0.05 and |log FC| > 2) of each of the two circRNA expression profiles were identified. To enhance the accuracy of the results, the DEcircRNAs were analyzed by Venn analysis using the FunRich software (version 3.1.3) [19], and only the intersections circRNAs between the GSE116617 and GSE94591 were calculated downstream. The threshold of *P* < 0.05 and |log FC| ≥ 1 were used to identify DEmiRNAs, and the threshold of *P* < 0.01 and |log FC| ≥ 2 were used for DEmRNAs.

### Construction of the circRNA-miRNA-mRNA network

To construct the circRNA-miRNA-mRNA network, we first predict the target miRNAs of DEcircRNAs from circbank database [20]. To increase the reliability of analysis, we selected the overlapping parts of predicted target miRNAs and the DEmiRNAs as the target miRNAs and established a circRNA-miRNA network. Next, we searched for the miRNA-predicted mRNAs pairs from the miRWalk database and the overlapping parts between the predicted mRNAs and the DEmRNAs were selected to construct a miRNA-mRNA network related to tumorigenesis. MiRWalk [21] database is a comprehensive predicted miRNA-targeted mRNA tool using the TarPmiR algorithm and two other miRNA-target prediction data-sets (TargetScan and miRDB). The TarPmiR algorithm applies a random-forest-based approach to predict miRNA target sites, and the score > 0.8 was set as the cut-off criterion in this study. The miRNA-mRNA pairs with the score > 0.8 and in both the two other miRNA-target prediction data-sets were selected for further study. Only the miRNAs in the miRNA-mRNA network were selected to construct the circRNA-miRNA-mRNA network. Finally, the visualized circRNA-miRNA-mRNA network was established using the Cytoscape software (version 3.7.1) [22]. The basic characteristics of the involved circRNAs were obtained from the Cancer-Specific CircRNA Database (CSCD) [23], including the number and the position of the microRNA response element, the open reading frame and the RNA binding protein.

### Gene ontology and pathway enrichment analysis

We performed the Gene Ontology (GO) annotation and KEGG pathway analyses [24, 25] of the DEmRNAs involved in the competing endogenous RNA network using the “clusterProfiler” package (version 3.16.1) [26] in R/Bioconductor. *P* values were corrected with Benjamini-Hochberg method. P.adjust value < 0.05 were considered as statistically significant.

### Construction of protein–protein interaction network and identification of key module

The protein–protein interaction (PPI) network was constructed using Search Tool for the Retrieval of Interacting Genes database (STRING, version 11.0) [27] based on the DEmRNA in the ceRNA network, and the unconnected nodes were removed. STRING is a database of known and predicted protein-protein interactions which covers 24,584,628 proteins from 5090 organisms. A comprehensive score for each protein-protein pair which distributes from 0 to 1 is used in the STRING, and the score > 0.4 was set as the cut-off criterion in this study. The Molecular Complex Detection (MCODE, version 1.6.1) [28], an application in the cytoscape, was used to screen the key modules from the PPI network, with the degree cut-off set to 5. CytoHubba [29], another application in the cytoscape, was used to find the hub genes from the PPI network. CytoHubba plugin uses 12 algorithms to evaluate the hub genes, and the top 20 genes which were found in at least 9/12 algorithms were considered as hub genes. The intersections between the genes in the key module and the hub genes screened by CytoHubba application were considered as hub genes.

### Validation and survival analysis of the hub genes

The GEPIA 2 [30] was used to validate the mRNA expression levels of the hub genes. GEPIA 2 compare the expression level between AML patients and normal controls from the TCGA and the GTEx projects, using a standard processing pipeline. Differentially expressed genes were considered as *P* value < 0.01. The survival curve was plotted in GEPIA 2 to explore the relationship between the overall survival and the hub genes. The quartile was used for group cutoff, and the P value < 0.05 was considered as significant.

## Results

### Differentially expressed circRNAs, miRNAs and mRNAs between patients and healthy controls

**Fig. 1:**
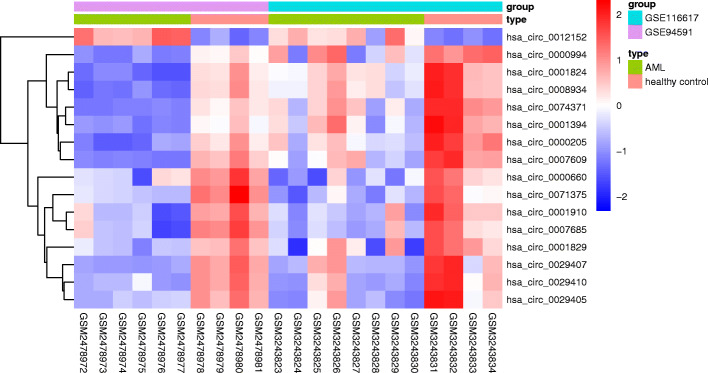
Heatmap of the fifteen differentially expressed circRNAs from the two microarray datasets

A total of 107 differentially expressed circRNAs (including 22 upregulated and 85 downregulated) in GSE116617 and 73 differentially expressed circRNAs (including 13 upregulated and 60 downregulated) in GSE94591 were identified. Fifteen co-downregulated circRNAs (hsa_circ_0000205, hsa_circ_0074371, hsa_circ_0001824, hsa_circ_0001829, hsa_circ_0001910, hsa_circ_0029410), hsa_circ_0007685, hsa_circ_0029407, hsa_circ_0000994, hsa_circ_0000660, hsa_circ_0029405, hsa_circ_0001394, hsa_circ_0007609, hsa_circ_0008934, hsa_circ_0071375) and one co-upregulated circRNA (hsa_circ_0012152) were identified (Fig. 1). A total of 125 DEmiRNAs (including 25 upregulated and 100 downregulated) and 629 DEmRNAs (including 141 upregulated and 488 downregulated) were identified. Among them, FLT3 and OLFM4 were the most upregulated and downregulated mRNAs; hsa-miR-181a-3p and hsa-miR-382-5p were the most upregulated and downregulated miRNAs, respectively.

### The circRNA-miRNA-mRNA network

**Fig. 2:**
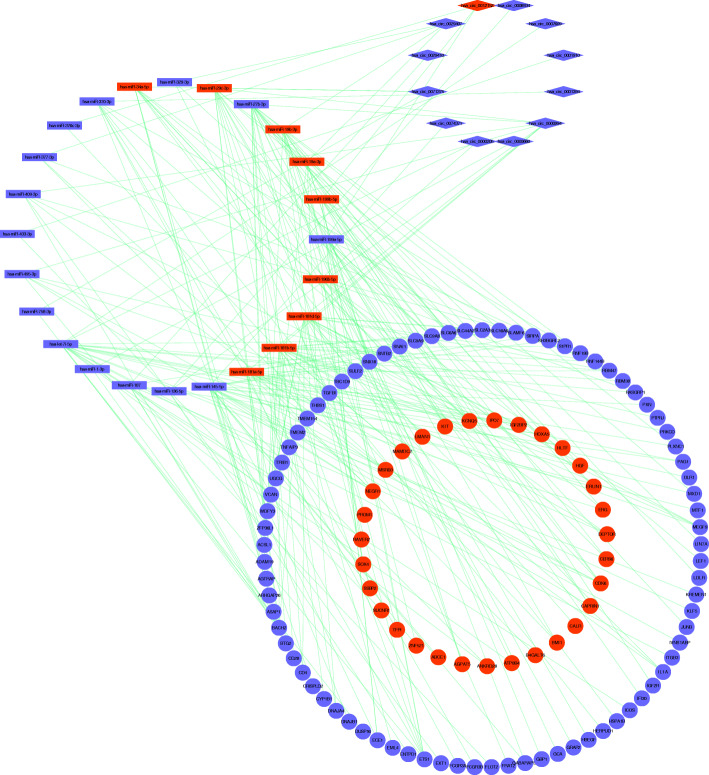
The circRNA-miRNA-mRNA network in the AML. Diamonds indicate circRNAs, rectangles indicate miRNAs, and ellipses indicate mRNAs. The red and blue nodes represent up-regulation and down-regulation, respectively

**Fig. 3:**
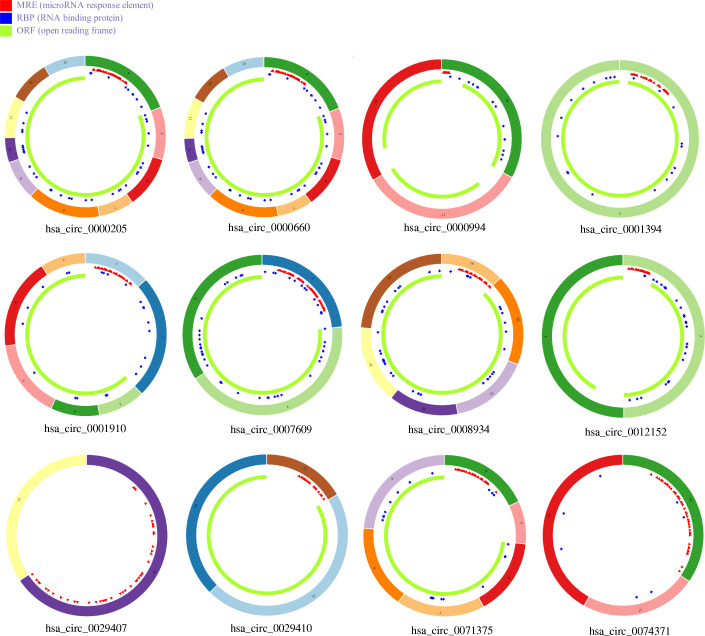
Basic structural patterns of the twelve circRNAs

A circRNA-miRNA-mRNA network was established and analyzed using Cytoscape v3.7.1. A total of 27 circRNA-miRNA pairs and 208 miRNA-mRNA pairs, including 12 circRNAs, 24 miRNAs and 112 mRNAs were included in the ceRNA network (Fig. 2). The basic characteristics of the 12 circRNAs were shown in Fig. 3.

### Enrichment analysis of the DEmRNAs involved in the ceRNA network

**Fig. 4:**
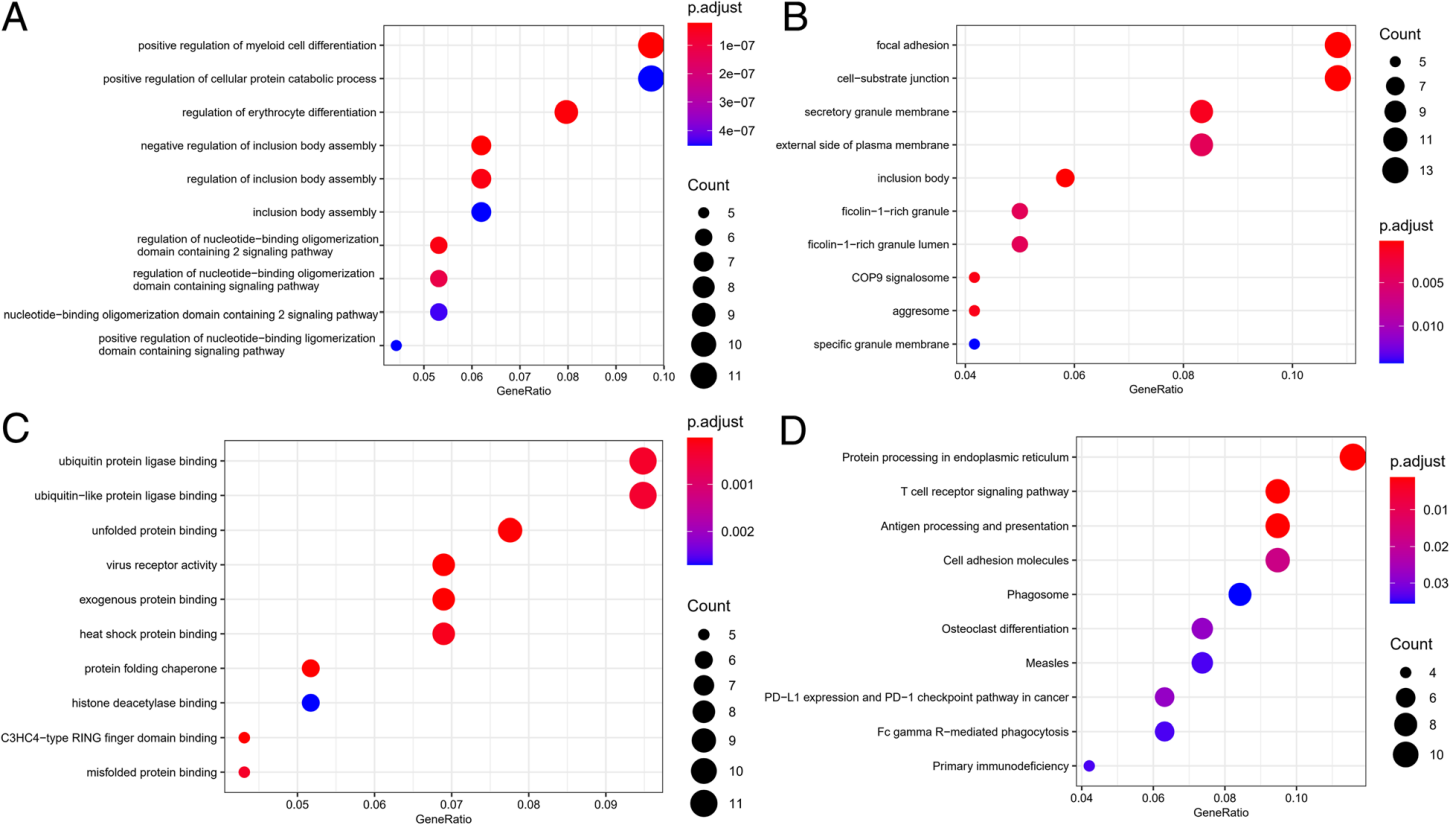
Significantly enriched top 10 GO terms and KEGG analysis pathways of DEmRNAs involved in ceRNA network. **a** biological process; **b** cellular component; **c** molecular function and **d** KEGG signaling pathways

To determine the candidate circRNA-mediated aberrant signaling pathways, the GO annotation and KEGG pathway analysis were performed to discover the aberrantly regulated biological process, cellular component, molecular function and signaling pathways of the tumorigenesis of AML. The GO analysis suggested that biological process, such as “negative regulation of inclusion body assembly” , “positive regulation of myeloid cell differentiation” and “regulation of erythrocyte differentiation” , cellular component, such as “inclusion body” , “focal adhesion” and “cell-substrate junction” , molecular function, such as “C3HC4-type RING finger domain binding” , “protein folding chaperone” and “virus receptor activity” were among the most enriched terms. And the “Protein processing in endoplasmic reticulum” , “Antigen processing and presentation” , “T cell receptor signaling pathway” were among the most enriched pathways according to the KEGG analysis. The top 10 GO terms and KEGG analysis pathways were shown in Fig. 4.

### PPI network and the key module

**Fig. 5:**
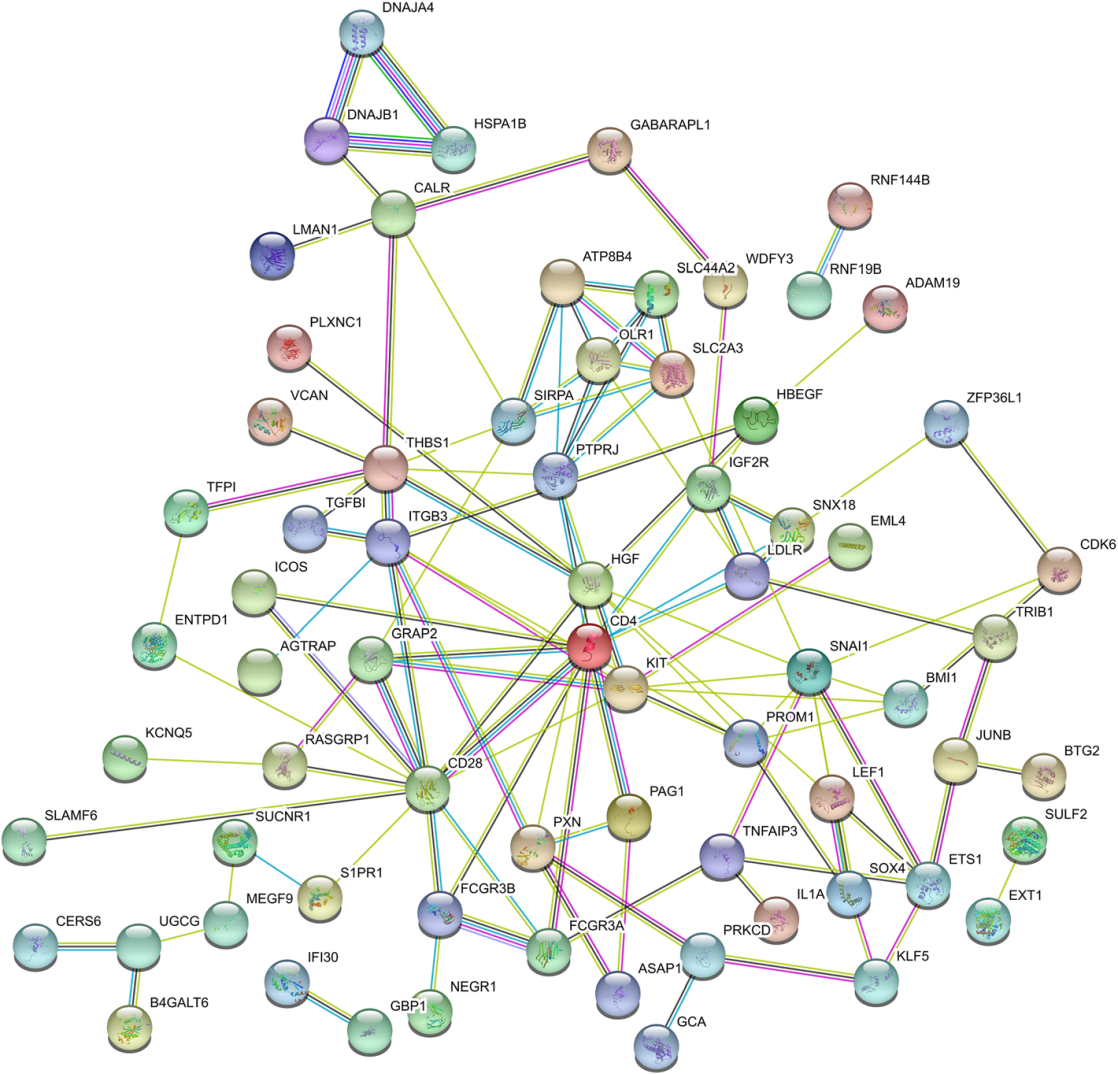
Protein-protein intersection (PPI) network analysis of DEmRNA involved in the ceRNA network

Based on the STRING database, the PPI network, including 70 genes and 120 pairs, was established to show the interaction of the target genes involved in the tumorigenesis of AML (Fig. 5). The MCODE application in cytoscape was used to find the key module, and a key module of 13 genes and 22 edges was identified. Then the top 20 genes, which were found in at least 9/12 topological algorithms, were found using CytoHubba application. The intersections between the key module and the top 20 genes were identified and considered as hub genes. Finally, we identified 6 hub genes which were potential related to the tumorigenesis of AML, namely CD28, FCGR3A, HGF, LDLR, PTPRJ and SIRPA. The circRNA-miRNA-hub gene subnetwork, including 4 circRNAs, 5 miRNAs and 6 mRNAs, were established based on related circRNA-miRNA-mRNA regulatory modules (hsa_circ_0074371 − hsa-miR-145-5p − SIRPA, hsa_circ_0029407 − hsa-miR-19b-3p − LDLR, hsa_circ_0001394 − hsa-miR-370-3p − FCGR3A, hsa_circ_0000994 − hsa-miR-27b-3p − CD28/HGF and hsa_circ_0000994 − hsa-miR-495-3p − HGF/PTPRJ).

### Validation and survival analysis of the hub genes

**Fig. 6:**
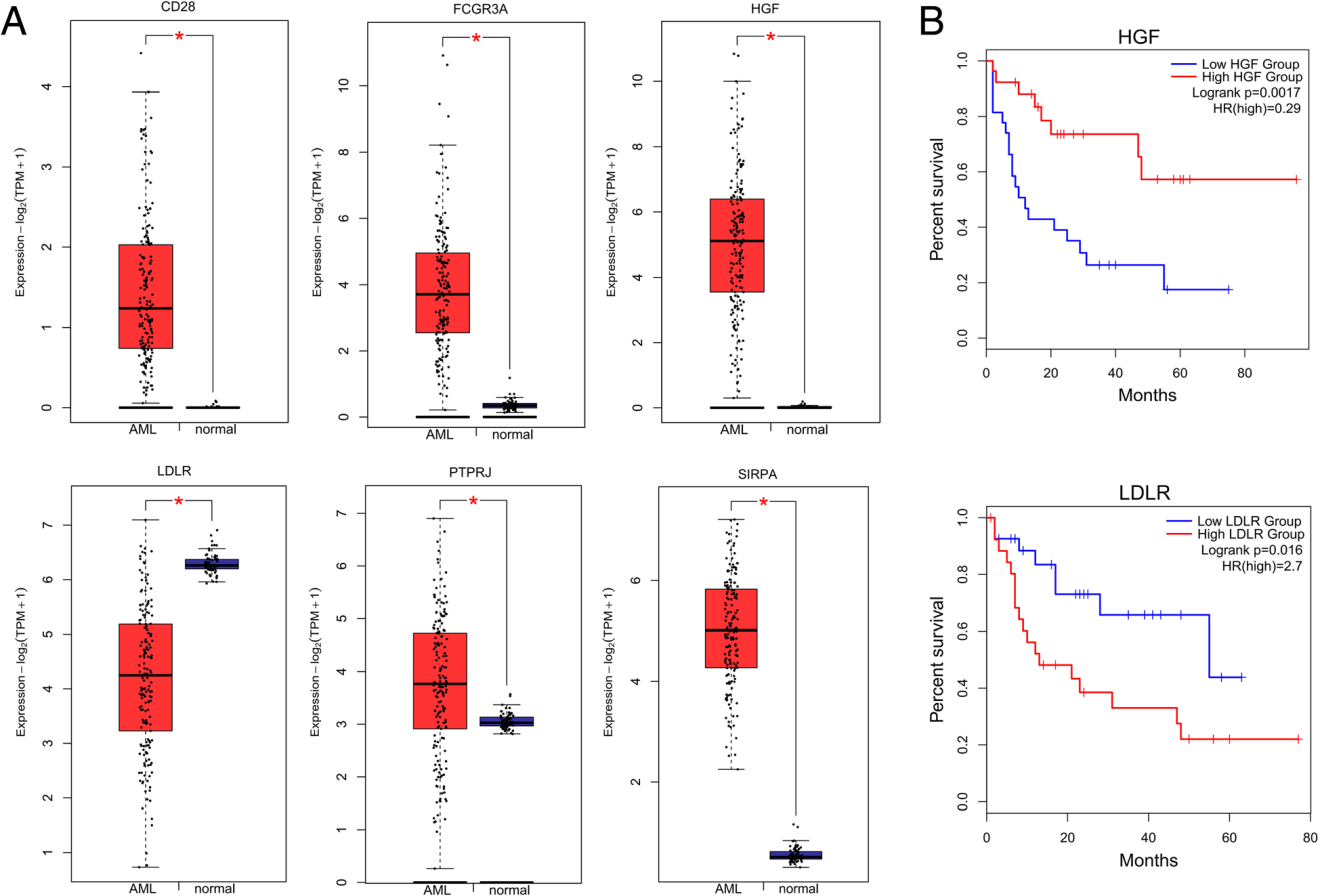
Validation and survival analysis of the six hub genes. **a** The mRNA expression level of six hub genes derived from TCGA database and GTEx projects. **b** The overall survival analysis of HGF and LDLR which are significantly related to the overall survival (OS) of patients with AML (*P* < 0.05)

To further validate the hub genes with more reliable supports, the GEPIA 2 was used to compare mRNA expression level of six hub genes derived from TCGA database and GTEx projects. As shown in Fig. 6a, the hub genes, CD28, FCGR3A, HGF, LDLR, PTPRJ and SIRPA were significant differentially expressed between AML patients and normal controls.

Next, we analyzed the overall survival (OS) to determine the prognostic value of the hub genes. The survival curves of HGF and LDLR were shown in Fig. 6b. Higher expression of HGF was associated with increased OS (HR = 0.29, *P* = 0.0031), and higher expression of LDLR was associated with poor OS (HR = 2.7, *P* = 0.016).

## Discussion

Acute myeloid leukemia is a complex hematological malignancy caused by dysregulation of various genes and signaling pathways [31, 32]. As a novel post-transcriptional gene regulated molecule, a growing number of researches have revealed the significance of circRNA in the pathogenesis of various complicated human diseases, including malignant tumors [33, 34]. CircRNA contains various MREs which could absorb and inhibit the activity of miRNAs and then, affect the expression of the downstream target mRNAs, and this function of circRNA is called miRNA sponge [35]. Nevertheless, the exact role of circRNA in the tumorigenesis of AML remains largely unknown. To identify the comprehensive role of circRNA as miRNA sponge in AML, we first screened the DEcircRNAs, DEmiRNAs and DEmRNAs from microarray data. After predicting the interactions between the RNAs based on biological predictions, a circRNA-miRNA-mRNA regulatory network was established. The mRNAs involved in the ceRNA network, which could be potentially regulated by circRNA, were used for GO annotation and KEGG pathway analysis. Then we constructed a PPI network model and the 6 hub genes (CD28, FCGR3A, HGF, LDLR, PTPRJ and SIRPA) were identified. These 6 hub genes were validated and survival analyzed using data from TCGA and the GTEx projects, which increase the accuracy of the ceRNA network. We also constructed a circRNA-miRNA-hub gene subnetwork based on related circRNA-miRNA-mRNA regulatory modules.

More and more studies revealed that dysregulation of circRNA is associated with pathogenesis of AML, suggesting the potential therapeutic targets and biomarkers of circRNA. A study [36] has shown that hsa_circ_0009910 highly expressed in AML patients, and decrease hsa_circ_0009910 expression level will inhibit AML cell proliferation and induced apoptosis. Zhang et al. [37] reported that hsa_circ_0000370 was markedly increase in AML patients and cell lines. The authors also found that overexpress or knockdown hsa_circ_0000370 level will significantly increase or decrease the viability of AML cell lines, respectively, and this function was implemented through sponging miR-1299 and increasing S100A7A expression. However, as little we know about circRNA, more research should be taken to explore the relationship between this novel molecule and pathogenesis of AML.

In the current study, we identified 12 circRNAs (hsa_circ_0000205, hsa_circ_0074371, hsa_circ_0001910, hsa_circ_0029410, hsa_circ_0012152, hsa_circ_0029407, hsa_circ_0000994, hsa_circ_0000660, hsa_circ_0001394, hsa_circ_0007609, hsa_circ_0008934, hsa_circ_0071375) involved in the ceRNA network. Three of these twelve circRNAs were reported associated with pathogenesis of tumor. Hsa_circ_0001910 was found significantly down regulated in breast cancer, and the expression of hsa_circ_0001910 was associated with breast cancer progression. Hsa_circ_0001910 acting as the miRNA sponge to regulate breast cancer growth and metastasis through hsa_circ_0001910 − miR-26b-3p/miR-660-3p − Ras signaling pathway axis [38, 39]. Another circRNA, hsa_circ_0000994, was found significantly downregulated in bladder cancer, and could suppress migration and invasion of bladder cancer cell via targeting miR-130b/miR-494 to enhance PTEN expression [40]. And hsa_circ_0008934 was found promoted the proliferation and migration of osteosarcoma cells through hsa_circ_0008934 − miR-145-5p − E2F3 axis [41]. None of the other 9 circRNAs were reported involved in the progression of cancer. Whether these circRNAs play a crucial role in the pathogenesis of cancer, especially AML, still remains unknown.

A total of 12 circRNAs, 24 miRNAs and 112 mRNAs were identified in the ceRNA network in this study, while some of them have been reported as biomarkers or therapeutics targets. To further identify and validate the key circRNAs, a circRNA-miRNA-hub gene subnetwork, including 4 circRNAs (hsa_circ_0074371, hsa_circ_0029407, hsa_circ_0001394 and hsa_circ_0000994), 5 miRNAs (miR-145-5p, miR-19b-3p, miR-370-3p, miR-27b-3p and miR-495-3p) and 6 hub genes (CD28, FCGR3A, HGF, LDLR, PTPRJ and SIRPA), was constructed. All of the hub genes identified in the subnetwork were previously reported correlated with tumorigenesis of leukemia. CD28, binding with co-stimulatory molecules in antigen presenting cells (APCs), could be a potential immunotherapy target for AML [42, 43]. FCGR3A, as part of the receptor of IgG, could mediate antibody dependent cell-mediated cytotoxicity by binding CD96 to against the AML stem cell [44]. As one of the growth factors secreted by bone marrow stromal cell, HGF promotes proliferation and migration of AML cell through PI3K-AKT and MAPK/ERK signaling pathway [45, 46]. The expression of LDLR in AML patients is higher than healthy controls, and higher HGF expression is associated with lower overall as well as event-free survival and higher cumulative incidence of relapse, suggesting the prognostic value of LDLR [47]. PTPRJ, a type of protein-tyrosine phosphatases, attenuates AML cell transformation through negatively regulating FLT3 expression and could be a candidate target for therapy [48, 49]. SIRPA, the CD47 receptor primarily expressed in macrophage, has been demonstrated the therapeutic target for macrophage-mediate elimination of AML in both vitro and vivo [50–52]. In a word, previous studies confirmed the ceRNA we constructed participate in the pathogenesis of AML and could be the effective diagnostic and therapeutic targets. As our results were mainly gained from bioinformatics model, further studies are crucial to explore the exact role of these circRNAs in the tumorigenesis of AML in depth.

## Conclusions

This work investigated the differentially expressed circRNAs, miRNAs, and mRNAs in AML and constructed a circRNA-miRNA-mRNA network. The circRNA-miRNA-hub gene subnetwork revealed four key circRNAs which would participate in the pathogenesis of AML, suggesting novel potential prognostic, diagnostic and therapeutic strategy for AML.

## Data Availability

The datasets analyzed during the current study are available via the Gene Expression Omnibus web portal (https://www.ncbi.nlm.nih.gov/geo/, accession number: GSE116617, GSE94591, GSE142699 and GSE114868).

## Abbreviations

AML: Acute myeloid leukemia
GEO: Gene Expression Omnibus
ceRNA: Competing endogenous RNA
HSC: Hematopoietic stem cell
OS: Overall survival
ncRNA: Noncoding RNA
MREs: microRNA response elements
DEmRNAs: Differentially expressed mRNAs
DEmiRNAs: Differentially expressed miRNAs
DEcircRNAs: Differentially expressed circRNAs
GO: Gene ontology
KEGG: Kyoto Encyclopedia of Genes and Genomes
TCGA: The Cancer Genome Atlas
GTEx: Genotype-Tissue Expression
PPI: Protein–protein interaction
